# Beasts of the Earth: Animals, Humans, and Disease

**DOI:** 10.3201/eid1107.050527

**Published:** 2005-07

**Authors:** Nina Marano

**Affiliations:** * Centers for Disease Control and Prevention, Atlanta, Georgia, USA

**Keywords:** zoonoses, emerging disease, risk factor

In their new book Beasts of the Earth: Animals, Humans, and Disease, Torrey and Yolken provide us with a thoroughly researched and well-written account of the animal origins of many human diseases. With interesting insight into this very timely topic, the authors describe the impact of animals and their diseases on the rise and subsequent decline of ancient civilizations. The book traces the history of humans' relationship with animals as humans evolved from hunters to villagers, traders, and, more recently, pet owners and international consumers, an evolutionary process that has given microbes unlimited passports to new populations and geographic areas. As testament to the adaptability of these pathogens, the book describes the increasing challenges to global health presented by zoonotic infections recently emerging from parts of Asia and Africa.

Fortunately, Torrey and Yolken also have plenty of advice for how the scientific community can address these challenges. The authors strongly emphasize the need for closer coordination and communication between the medical and veterinary sectors at national and international levels. Stressing that it is illogical for the animal and human health worlds to conduct zoonotic disease research separately, they state that interdisciplinary zoonotic disease research centers should be the model research platforms for the future.

Those who have read the 2003 Institute of Medicine Report Microbial Threats to Health: Emergence Detection and Response (Institute of Medicine, 2003) may find many of the themes in this book familiar. However, as the authors chillingly state, "We live with these pathogens in a negotiated peace, but what happens when biological circumstances change?" Torrey and Yolken do an excellent job of reminding us of the interconnectedness between human and animal hosts and their pathogens and environments.

